# Simultaneous measurements of foveal and peripheral aberrations with accommodation in myopic and emmetropic eyes

**DOI:** 10.1364/BOE.438400

**Published:** 2021-11-09

**Authors:** Dmitry Romashchenko, Petros Papadogiannis, Peter Unsbo, Linda Lundström

**Affiliations:** 1 Department of Applied Physics, Royal Institute of Technology, Stockholm, 11421, Sweden; 2 Current address: R&D, Johnson & Johnson Vision, Groningen, 9728 NX, The Netherlands

## Abstract

The difference in peripheral retinal image quality between myopic and emmetropic eyes plays a major role in the design of the optical myopia interventions. Knowing this difference under accommodation can help to understand the limitations of the currently available optical solutions for myopia control. A newly developed dual-angle open-field sensor was used to assess the simultaneous foveal and peripheral ( ${\mathrm{20}}^{\circ }$ nasal visual field) wavefront aberrations for five target vergences from -0.31 D to -4.0 D in six myopic and five emmetropic participants. With accommodation, the myopic eyes showed myopic shifts, and the emmetropic eyes showed no change in RPR. Furthermore, RPR calculated from simultaneous measurements showed lower intra-subject variability compared to the RPR calculated from peripheral measurements and target vergence. Other aberrations, as well as modulation transfer functions for natural pupils, were similar between the groups and the accommodation levels, foveally and peripherally. Results from viewing the same nearby target with and without spectacles by myopic participants suggest that the accommodative response is not the leading factor controlling the amplitude of accommodation microfluctuations.

## 1. Introduction

Myopia is a growing global problem, that also carries increased risks for severe ocular conditions [1–4]. Therefore, it is important to develop methods for stopping myopia onset and development. The most effective myopia interventions that involve optics are multifocal or progressive spectacles, orthokeratology, and multifocal contact lenses [5,6]. However, the treatment mechanisms of these optical interventions are not fully understood yet and their myopia prevention effect appears to vary between individuals [5,6].

One common feature of the optical myopia interventions is that they all, either by design or as a side effect, change the optical image quality on the peripheral retina compared to conventional spectacles or soft contact lenses [7–10]. Previously, studies on monkeys have shown that a hyperopic peripheral defocus can cause the eye to grow myopic [11,12]. Therefore, it is likely that the change in the peripheral retinal image induced by the interventions is part of their treatment mechanism. The varying magnitude of the treatment effect, however, shows a need for a more comprehensive knowledge of peripheral retinal image quality, especially in close-to-real-life conditions. These include (but are not limited to) binocular viewing, different accommodation demands, and time-resolved measurements that would account for temporal variations in the ocular optics, such as accommodation microfluctuations.

Current knowledge of the peripheral aberrations during accommodation is limited. Most previous work studied peripheral refractive errors [13–19], and only a few studied full retinal image quality [20–23]. Furthermore, the studies on peripheral refractive errors do not agree between each other. They report either no change [13,18–21,23], myopic shift [14,15,21] or hypermetropic shift [14,16,17] in relative peripheral refraction (RPR) with accommodation for myopic and emmetropic eyes. Such a diversity may be a consequence of monocular accommodation stimulation without accommodation state tracking. Studies on monocular accommodation in myopic eyes have found up to 1.0 D of accommodative lag for a 4.0 D accommodative demand [24–27]. This might have a significant influence on the estimated RPR. Simultaneous foveal and peripheral wavefront measurements allow for direct assessment of accommodative lag and thus more accurate RPR calculation. Therefore, such measurements, performed for several steps of accommodation, can enrich the knowledge on peripheral ocular aberrations and myopia development.

In this study foveal and peripheral aberrations are measured at the same time, with known accommodation states, so that one can be more confident about the accuracy of findings regarding differences between foveal and peripheral aberrations than those of previous studies. This work has three main objectives. First, to see which of the previous results on RPR can be verified with this experimental setting. Second, to investigate if there is any difference in peripheral image quality for myopic participants with and without spectacles when viewing nearby targets. And third, to study the temporal aspect of the eye’s optics as foveal and peripheral accommodation microfluctuations and compare them across the conditions. For these purposes, we use a recently developed dual-angle open-field wavefront sensor [28]. The experiments consist of synchronized, time-resolved foveal-peripheral wavefront measurements for different accommodation states in emmetropic and myopic eyes.

## 2. Methods

### 2.1 Measurements

This study was carried out according to the tenets of the Declaration of Helsinki and was approved by the Swedish Ethical Review Authority. Before the experiment, each participant was provided with a detailed description of the measurement process and the purpose of the experiment. A written consent was obtained from every participant.

Wavefront measurements of the right eye were performed simultaneously at fovea and at ${\mathrm{20}}^{\circ }$ nasal visual field (VF) angle, using a dual-angle open-field wavefront sensor. This device consists of two measurement channels. Each channel has a Hartmann-Shack wavefront sensor, which enables data acquisition at two VF locations at the same time. The configuration of the device allows a participant to view the targets binocularly through a hot mirror, thus creating natural accommodation conditions. A more detailed description of the device, including the post-processing routines, is provided elsewhere [28].

During the experiments, the participants viewed a brightly illuminated (photopic conditions) black-and-white picture subtending $2^{\circ }$ in diameter and containing fine details (angular frequencies up to 50 cycles/degree). The ambient light in the experimental room was dimmed to minimize distractions. The participants were instructed to look at the picture and try to see it as sharply as possible. The focusing target was successively placed at five different distances along the line of sight of the right eye corresponding to the following target vergences: -0.31 D, -1.0 D, -2.0 D, -3.0 D, and -4.0 D. The linear dimensions of the target were scaled for each viewing distance so that the target always subtended $2^{\circ }$ of the VF. During the measurements, the myopic group wore habitual spectacle corrections. Additionally, the myopic group was measured uncorrected for the same -4.0 D target as in the spectacle correction case (0.25 m away from the eye).

Each measurement set lasted 30 seconds and three repetitions were made for each condition. In order to perform the measurements as quickly as possible, there was no randomization in experimental conditions. To further minimize fatigue, the participants were given several minutes to rest in between each target change.

### 2.2 Participants

Seven habitually-emmetropic and six myopic persons participated in this study. The measurements for two emmetropic participants were excluded: one due to the distortion of the spotfield image by the eyelashes, and one due to a constant accommodative lag of approximately 1.0 D for all target vergences. The final sample thus consisted of five emmetropic and six myopic eyes.

The ages of the five habitual emmetropic participants were 24-29 years, and the unaided visual acuity was at least 1.0 (20/20). The spherical equivalents (SEs) at spectacle plane, verified with subjective refraction, were 0 D, +0.125 D, +0.25 D, -0.875 D, and -0.375 D.

The ages of the six myopic participants were 24-30 years. The SEs at spectacle plane, calculated from prescriptions, were -3.875 D, -2.375 D, -1.25 D, -1.25 D, -1.5 D, and -2.875 D. For both refractive groups, the cylindrical power error was less than 0.75 D. None of the participants had reported any other ocular disorders.

### 2.3 Data analysis

Zernike coefficients up to the $6^{th}$ order were obtained from spotfield image processing using the algorithm proposed by Lundström and Unsbo [29]. The measurement wavelength was 830 nm, and the Zernike coefficients were recalculated to 550 nm wavelength as a part of post-processing [30]. The foveal and peripheral modulation transfer functions (MTFs) were calculated according to Romashchenko et al. [31]: averaged over all pupil meridians and additionally averaged over the time of the experiment. The ellipticity of the pupil for off-axis MTF calculations was taken into account by a cosine function [32].

The refractive errors were calculated from Zernike coefficients using the following formulas [33]:

(1) $$SE[D]=-\frac{4\sqrt{3}}{r_{p}^{2}}\cdot c_{2}^{0}+\frac{12\sqrt{5}}{r_{p}^{2}}\cdot c_{4}^{0}-\frac{24\sqrt{7}}{r_{p}^{2}}\cdot c_{6}^{0}+\cdots \text{,}$$

(2) $$J_{0}[D]=-\frac{2\sqrt{6}}{r_{p}^{2}}\cdot c_{2}^{2}+\frac{6\sqrt{10}}{r_{p}^{2}}\cdot c_{4}^{2}-\frac{12\sqrt{14}}{r_{p}^{2}}\cdot c_{6}^{2}+\cdots \text{ ,}$$

(3) $$J_{45}[D]=-\frac{2\sqrt{6}}{r_{p}^{2}}\cdot c_{2}^{-2}+\frac{6\sqrt{10}}{r_{p}^{2}}\cdot c_{4}^{-2}-\frac{12\sqrt{14}}{r_{p}^{2}}\cdot c_{6}^{-2}+\cdots \text{ ,}$$

where SE is the spherical equivalent, $J_{0}$ and $J_{45}$ are the Jackson cross-cylinders for expressing astigmatism, $c_{n}^{m}$ are the corresponding Zernike coefficients, and $r_{p}$ is the radius of the pupil.

Accommodation microfluctuations (AMFs) were represented as the standard deviation of the time-resolved SE for each measurement set. Thus, for a given person and accommodation demand, the AMFs are an average of these standard deviations throughout the three repetitions.

The measurements at $20^{\circ }$ nasal VF were performed through the spectacles of the corrected myopic participants. This means that the value of the off-axis measurement angle is subject to spectacle magnification. However, in this study the spectacle magnification for the myopic participants was too low to have any substantial effect. Deviations from the $20^{\circ }$ were only between ${\mathrm{-0.15}}^{\circ }$ and ${\mathrm{-0.74}}^{\circ }$ .

The spectacles also imposed a difference between the measured and the real ocular accommodation of the myopic eyes. The accommodative response was therefore calculated using the intermediate image (from the measured wavefront curvature when imaged through the spectacles). In this paper we use the term ’accommodation increase’ which was calculated for each participant as foveal SE at the tested target minus foveal SE at the 0.31 D target. This way we aim to bypass the differences between the lowest accommodation demand (0.31 D) and the location of the far point of the studied eyes. For myopic eyes, this accommodation increase was calculated by taking the spectacle magnification into account. That is, first finding an intermediate image location (after the spectacles) from the measured SE, and then calculating the accommodation increase using this intermediate image location.

## 3. Results

**Fig. 1.**
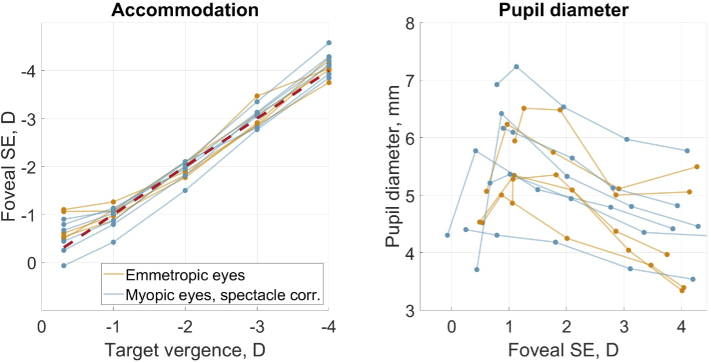
Left: foveal SE as a function of target vergence. As a reference, the red dashed line shows foveal SE equal to target vergence. Right: pupil diameter as a function of foveal SE. For myopic eyes the spectacle magnification was taken into account. In both figures emmetropic eyes are depicted with yellow lines, myopic eyes with spectacle correction are depicted with blue lines, and the dots show the measured values.

Foveal SE for different target vergences and pupil diameters are presented in Fig. 1. Yellow lines correspond to emmetropic eyes, blue lines correspond to myopic eyes with spectacle correction, and the dots represent the measured values. As accommodation demand increases, SE becomes more negative (Fig. 1, left) and the pupil diameter becomes smaller (Fig. 1, right). It is worth noting that, even for -4.0 D of target vergence, all participants show very little accommodative lag. Table S1 (Supplement 1) summarizes data depicted in Fig. 1, left.

**Fig. 2.**
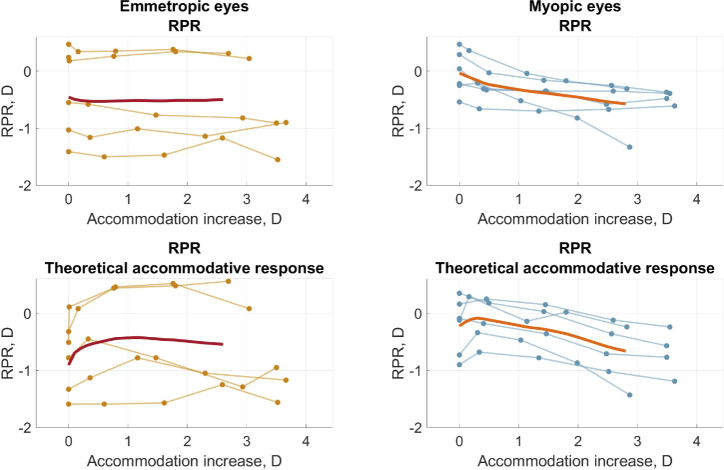
RPR as a function of accommodation increase for fovea and ${\mathrm{20}}^{\circ }$ nasal VF. Top row shows RPR from unaltered foveal and peripheral data. The bottom row curves were obtained from the same peripheral data but using the theoretical foveal accommodative response for the tested target. Left column represents the emmetropic group, and right column represents the myopic group with spectacle correction. Thin lines correspond to individual eyes, dots show the measured values, and bold lines depict average curves.

**Fig. 3.**
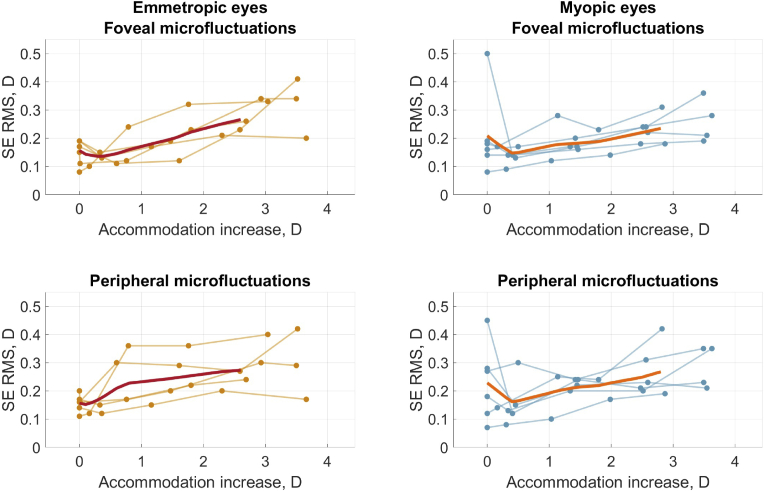
Accommodation microfluctuations (RMS of the time-variation in SE) as a function of accommodation increase for fovea and ${\mathrm{20}}^{\circ }$ nasal VF. Left column represents emmetropic group, and the right column represents myopic group with spectacle correction. Thin lines correspond to individual eyes, dots show the measured values, and bold lines show average curves.

The changes of optical aberrations with accommodation were studied separately for emmetropic and myopic participants. In Fig. 2 the top row depicts the RPR curves calculated from simultaneous foveal-peripheral measurements. For comparison, the bottom row shows RPR where the foveal SE is substituted with the theoretical accommodative response for the tested target. It can be seen that when the foveal measurements are included (top row), the intra-subject variability of the RPR is lower. For the myopic eyes, the RPR shifts to more negative values with accommodation. For the emmetropic eyes, the RPR has larger inter-subject variation and no noticeable difference between accommodative states. Figure S1 (Supplement 1) represents astigmatism ${\text{J}}_{0}$ and ${\text{J}}_{45}$ for fovea and periphery. Both astigmatisms undergo only minor, non-significant changes with accommodation, which is in line with previous work [17,20,21,34]. Figure 3 illustrates the AMFs amplitude for the central and peripheral VF angles. The amplitude is growing as the accommodation increases. In all of the mentioned figures, the left-column graphs correspond to the emmetropic group and the right-column graphs correspond to the myopic group with spectacle correction.

The differences of the ocular optical errors between 0.31 D and 4.0 D accommodative targets were analyzed using the Wilcoxon signed rank test on paired samples. Significance level was set to α=0.05 for a two-tailed test. Note that for n = 5 the Wilcoxon test cannot reach significance level of 0.05 [35]: the lowest possible p-value is 0.06, which corresponds to the critical value of 0 (Mathworks Matlab, ver. R2019a). Results for the refractive errors are given in Table 2. The myopic shift in RPR of the myopic group is statistically significant with p=0.03 . Wavefront aberrations are summarized as Zernike coefficients up to the $4^{th}$ order in Tables S2 and S3 (Supplement 1). In the emmetropic eyes, the Wilcoxon test showed statistically significant difference with accommodation in foveal $C_{4}^{-4}$ , foveal $C_{4}^{4}$ , and peripheral $C_{4}^{-2}$ . In the myopic eyes, statistical significant difference was found in peripheral $C_{3}^{3}$ and peripheral $C_{4}^{0}$ .

**Fig. 4.**
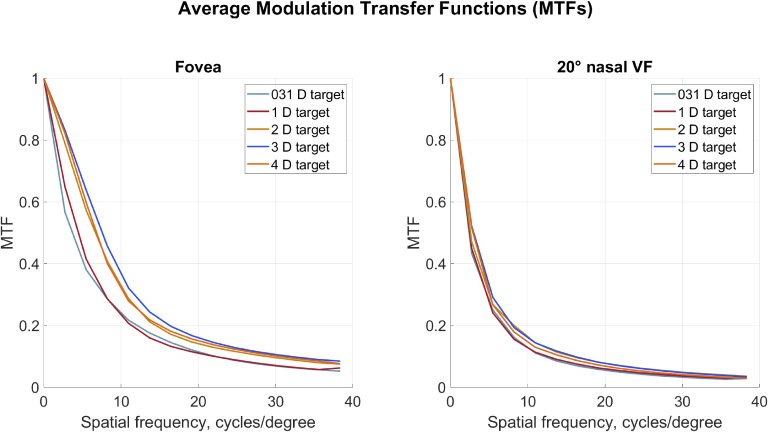
Average MTFs for fovea and ${\mathrm{20}}^{\circ }$ nasal VF for emmetropic eyes (n = 5). The curves are calculated using Zernike coefficients up to the $6^{th}$ order for wavelength 550 nm and natural pupil size. Effect of the target vergence was compensated prior to the calculations.

**Fig. 5.**
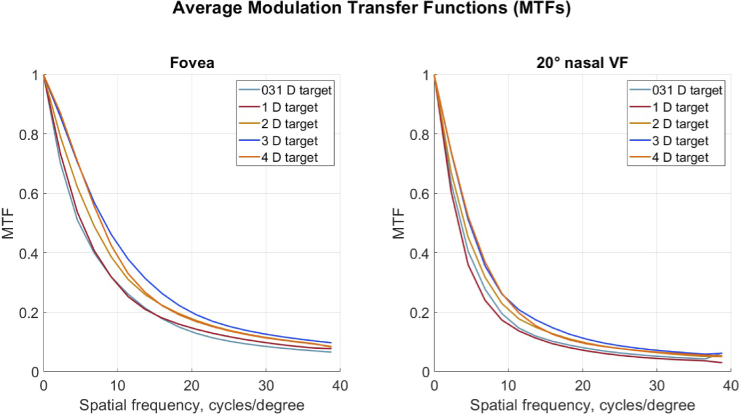
Average modulation transfer functions for fovea and $20^{\circ }$ nasal VF for myopic eyes with spectacle correction (n = 6). Other details are as for Fig. 4.

Average MTF curves for fovea and ${\mathrm{20}}^{\circ }$ nasal VF are depicted in Fig. 4 for emmetropic eyes and Fig. 5 for myopic eyes. The aim of these curves is to represent the actual monochromatic image quality, created on the retina for each focusing target. Therefore, the MTFs are calculated for the Zernike coefficients up to the $6^{th}$ order at the wavelength 550 nm for the natural pupil diameter. That is, the pupil size varied for different subjects and conditions (see Fig. 1, right). Furthermore, prior to the calculations, compensation for the target vergence was made. Zernike coefficients for the given pupil size, corresponding to a spherical wavefront converging to the foveal target location, were subtracted from each measured foveal and peripheral Zernike coefficients set. In Figs. 4 and 5 small changes in foveal MTF with accommodation can be seen: higher accommodative demands correspond to somewhat better MTF values. This trend is less pronounced in the peripheral MTFs. Additionally, Supplement 1 (Figure S2) contains MTFs for 3 mm pupil diameter with compensation for the target vergence.

The wavefront measurements of the myopic group were also compared between the cases of no correction and spectacle correction when viewing the target located 0.25 m (-4.00 D). The Wilcoxon signed rank test was performed on the paired data between the conditions. The results are summarized in Table 2 for pupil diameter and refractive errors and in Table S4 (Supplement 1) for the Zernike coefficients up to the $4^{th}$ order. The only statistically significant differences were found in foveal and peripheral $C_{4}^{0}$ and foveal $C_{2}^{2}$ (Table S4, Supplement 1).

## 4. Discussion

This study investigated simultaneous foveal-peripheral wavefront aberrations to obtain a more comprehensive picture of the optical quality with accommodation in myopic and emmetropic eyes. The sample groups in this work were small: five emmetropic and six myopic participants. Therefore, the purpose of this paper is to use the dual-angle sensor to verify previous results on ocular image quality with accommodation, and the quantitative results should be interpreted with caution. One of the functionally-emmetropic participants had SE = -0.875 D. We have verified that, in comparison to the rest of the emmetropic group, there were no outliers originating from this participant.

For the spectacle correction conditions in myopic eyes, the peripheral measurements were performed at oblique angles through the spectacles. This means that the off-axis aberrations of the spectacles are included in the measurements. For the aberration analysis, this offset is largely compensated for as we are analyzing the changes with accommodation rather than the absolute values. For the MTF analysis, it means that the curves are representing the habitual monochromatic image quality experienced by the eyes.

### 4.1 Accommodation and pupil size

The binocular open field of view of the dual-angle wavefront sensor provides participants with more natural conditions than monocular viewing. As a result, Fig. 1, left shows that all eyes, irrespective of the refractive group, do not experience any prominent accommodative lag. Furthermore, in agreement with earlier work [36,37], there is some accommodative lead present for the furthest target, and the pupil diameter decreases for higher accommodative responses.

For the majority of the participants, the size of the pupil was unexpectedly small for the 0.31 D target which cannot be accounted for by the accommodative lead (Fig. 1). This can probably be attributed to the preparation time for the experiment and the fact that the 0.31 D target was the first to test for all participants. As all participants were unfamiliar with the experiment, the adjustment procedures took about five minutes. During this time, the participants were supposed to constantly view the target with no ambient light in the room, which created some fatigue. This fatigue could explain the smaller pupil diameter and cause a larger accommodative lead, that is, accommodation moving more towards the resting state (even the emmetropic eye with SE = -0.875 D was accommodating away from its far point). The subsequent measurements were performed with small breaks and had much shorter adjustment times, which led to more alert participants and elevated pupil diameter values (Fig. 1, right).

### 4.2 More myopic RPR for myopic eyes with accommodation

Our conclusion from the measured RPR with accommodation state tracking is that it became more consistent with less fluctuations for the individual eyes. The RPR results in this study are in agreement with previous work reporting more myopic RPR for myopic eyes under accommodation [17]. Furthermore, the studies by Tabernero and Schaeffel [13], Calver et al. [18], Liu et al. [19], Mathur et al. [20], and Liu and Thibos [23] also found no change in RPR for emmetropic eyes with increasing accommodation.

As expected, comparison of the spectacle correction and no correction cases for the near-sighted group showed a slight but non-significant increase in RPR (Table 2), which can be attributed to the difference in accommodative responses between the conditions.

### 4.3 Small changes in higher-order aberrations with accommodation

Earlier studies on the variation in higher-order aberrations (HOA) with accommodation report that foveal spherical aberration becomes more negative with increasing accommodative response [20,22,38,39]. In the current study, this trend was small, observed only in the myopic group, and was statistically significant for the peripheral VF but not for the foveal VF (Table S3, Supplement 1). A similar negative shift in foveal and peripheral $C_{4}^{0}$ was also found when comparing the myopic eyes with and without spectacle correction (Table S3, Supplement 1). Such a shift is expected since spectacle correction is associated with higher accommodation. Apart from $C_{4}^{0}$ , peripheral $C_{3}^{3}$ in the myopic group also showed some change with accommodation. This also agrees with previous work [21]. The rest of the HOA Zernike coefficients show little differences, and the magnitudes of these differences are of the same order as reported in similar studies [20–22].

### 4.4 Little difference in retinal image quality with accommodation

As reported earlier, due to aberrations peripheral optical quality is worse than the foveal one [31,40–42] (see Figs. 4 and 5). The quality of the ocular optical system with natural pupil size did not seem to suffer from the change in target vergence. A small difference in retinal image quality with accommodation was also found in previous studies. For example, Cheng et al. concluded that "...a typical eye will benefit over the entire accommodative range (from 0 to 6 D) if aberrations are corrected for distance viewing." [22]. In the current study, the worsening of the MTF for emmetropic participants at 0.31 D target agrees with the larger accommodative lead for two members of that group (Fig. 1).

On average, the myopic group shows similar MTFs for the natural pupil diameters as the emmetropic group for all target vergences. It is also interesting to note that even though the RPR changes under accommodation for the myopic group, this change does not seem to have a negative effect on the peripheral MTFs. This indicates a large depth of field. With this in mind, it can be speculated that presence of additional aberrations, for example from multifocal lenses [10,43], may worsen the peripheral optical quality beyond that in emmetropic eyes. However, out-of-focus objects can still yield different image quality for myopic and emmetropic eyes across the VF [44]. Naturally, the retinal image quality will also depend on the complexity of the visual scene with various amounts of in- and out-of-focus objects [45].

### 4.5 AMFs: effect of pupil diameter overrides accommodative response

Figure 3 and Table 1 show that the magnitude of foveal AMFs, as expected [46], increases with increasing accommodation. Peripheral AMFs are lower in magnitude than the foveal ones but follow the same trend. Interestingly, the AMFs magnitude for uncorrected and spectacle corrected myopic eyes when viewing the -4.0 D target did not show any significant differences. The slightly higher magnitude for the spectacle correction case may be because of the spectacle magnification (would at maximum give a difference of 5%). This is surprising as the spectacle correction case is associated with higher accommodation levels. Thereby, the results presented in Table 1 and 2 of this study show how the AMFs can increase with accommodation in one experiment and stay unchanged in another for the same eyes. One interesting difference between the two experiments is the pupil size: it reduced with accommodation for the experiment in Table 1 but stayed unchanged between the two conditions in Table 2. Earlier studies have also reported elevated levels of AMFs for smaller artificial pupils at the same level of accommodation [47,48]. If this is the case, it supports the hypothesis mentioned by Charman and Heron [46] that the AMFs depend on the instantaneous depth of field, and that the increase in AMFs with accommodation is a consequence of the naturally occurring pupil constriction.

**Table 1.** Wilcoxon signed rank test results for the refractive error differences between 0.31 D and 4.0 D accommodation targets in emmetropic and myopic eyes with spectacle correction. Peripheral data correspond to the ${\mathrm{20}}^{\circ }$ nasal VF. Statistically significant differences are marked with bold font. Note that for the emmetropic group (n = 5) the lowest achievable p-value is 0.06 (critical value of 0). Additionally, for each parameter the average ± standard deviation for 0.31 D and 4.0 D targets is given. Note that the standard deviation here shows the variations of the average values between participants (not related to AMFs).

Parameter	p-value	Average for 0.31 D	Average for 4.0 D
	Emmetropic eyes, n = 5		
Relative Peripheral Refraction, D	0.31	-0.456 ± 0.805	-0.566 ± 0.804
Foveal ${\text{J}}_{0}$ , D	0.44	+0.102 ± 0.211	+0.158 ± 0.248
Foveal ${\text{J}}_{45}$ , D	0.31	-0.104 ± 0.098	-0.174 ± 0.214
Peripheral ${\text{J}}_{0}$ , D	0.44	-0.686 ± 0.268	-0.782 ± 0.183
Peripheral ${\text{J}}_{45}$ , D	0.13	-0.006 ± 0.294	+0.126 ± 0.373
Foveal Microfluctuations, D	0.06	+0.156 ± 0.046	+0.308 ± 0.080
Peripheral Microfluctuations, D	0.06	+0.156 ± 0.034	+0.304 ± 0.106
**Myopic eyes, spectacle correction, n = 6**			
Relative Peripheral Refraction, D	**0.03**	-0.035 ± 0.375	-0.582 ± 0.381
Foveal ${\text{J}}_{0}$ , D	0.06	+0.137 ± 0.174	+0.343 ± 0.236
Foveal ${\text{J}}_{45}$ , D	0.88	+0.047 ± 0.132	+0.063 ± 0.094
Peripheral ${\text{J}}_{0}$ , D	0.31	-0.212 ± 0.160	-0.278 ± 0.224
Peripheral ${\text{J}}_{45}$ , D	0.31	+0.287 ± 0.147	+0.333 ± 0.116
Foveal Microfluctuations, D	0.44	+0.208 ± 0.148	+0.255 ± 0.073
Peripheral Microfluctuations, D	0.44	+0.228 ± 0.136	+0.292 ± 0.094

**Table 2.** Wilcoxon signed rank test results for the pupil diameter and refractive errors differences between no correction and spectacle correction in the myopic eyes when viewing the target at 0.25 m. Peripheral data correspond to the $20^{\circ }$ nasal VF. Additionally, for each parameter the average ± standard deviation is given for spectacle correction and no correction conditions. Note that the standard deviation here shows the variations of the average values between participants (not related to AMFs).

Parameter	p-value	Average for no correction	Average for spectacle correction
	Myopic eyes, n = 6		
Foveal Pupil Diameter, mm	0.84	4.539 ± 0.849	4.578 ± 0.74
Foveal Mean Sphere, D	0.69	-4.242 ± 0.409	-4.158 ± 0.264
Relative Peripheral Refraction, D	0.66	-0.365 ± 0.663	-0.582 ± 0.381
Foveal Microfluctuations, D	0.75	0.245 ± 0.075	0.255 ± 0.073
Peripheral Microfluctuations, D	0.63	0.280 ± 0.138	0.292 ± 0.094

### 4.6 Limitations and future work

One of the limitations of the current study is the lack of the target vergence randomization when presenting the stimuli. Thus, the results, presented in this work, are potentially subject to selection bias. Additionally, the lengthy adjustment procedures for the 0.31 D target have led to constricted pupils and possibly some fatigue for that condition. Also, the intra-subject variability due to the somewhat small sample size may have masked some of the less pronounced trends. Finally, the span of accommodation demands used in this study is limited to 4 D due to the construction of the setup.

For the future work, it is suggested to increase the number of participants and studying a younger population group since myopia progresses mostly during adolescence. Experimentally, it is needed to add stimuli randomization and decrease initial adjustment time. In addition, including higher accommodative demands can also be of great interest. Some trends, such as accommodation microfluctuations, may not have a monotonous behavior throughout a large accommodation range [46].

## 5. Conclusion

This study investigated simultaneous foveal-peripheral wavefront aberrations under accommodation in myopic and emmetropic eyes. The continuous accommodation state tracking proved to be beneficial and gave more consistent trends in RPR with accommodation. The results suggest that RPR stayed the same for the emmetropic group and became more myopic for the myopic group with increasing accommodation. This supports some of the previous studies on the matter. Despite the differences in RPR, the MTFs for the emmetropic and the corrected myopic groups were similar. This goes in line with previous work that does not have unanimous conclusion whether the HOA differ between myopic and emmetropic eyes [49].

Additionally, comparison of the near-sighted eyes for spectacle correction and no correction viewing of the -4.0 D target revealed that the magnitude of AMFs may not be governed purely by the accommodative response. Instead, it appears to depend on the current depth of field which has also been hypothesized in earlier work.

For the future, there is a need for larger-scale studies with similar experimental settings that focus on young participants with progressing myopia and optical myopia interventions. We also suggest considering the overall image quality in addition to individual aberration terms.

## Data Availability

Data underlying the results presented in this paper are not publicly available at this time but may be obtained from the authors upon reasonable request.

## Supplemental document

See Supplement 1 for supporting content.
