# Supplementary material for: Simultaneous measurements of foveal and peripheral aberrations with accommodation in myopic and emmetropic eyes
Source: Biomed Opt Express. 2021 Nov 9;12(12):7422–33. doi: 10.1364/BOE.438400 (PMC8713693; doi:10.1364/BOE.438400)
Supplement: Supplementary file 1 [file boe-12-12-7422-s001.pdf]

Supplemental Document

Biomedical Optics EXPRESS

Simultaneous measurements of foveal and peripheral aberrations with accommodation in myopic and emmetropic eyes: supplement

DMITRY ROMASHCHENKO,[1,2,*] PETROS PAPADOGIANNIS,[1] PETER UNSBO,[1] AND LINDA LUNDSTRÖM[1]

[1] *Department of Applied Physics, Royal Institute of Technology, Stockholm, 11421, Sweden*
[2] *Current address: R&D, Johnson & Johnson Vision, Groningen, 9728 NX, The Netherlands*
[*] *dromashc@its.jnj.com*
https://www.aphys.kth.se/biox/research/vio

This supplement published with Optica Publishing Group on 9 November 2021 by The Authors under the terms of the Creative Commons Attribution 4.0 License in the format provided by the authors and unedited. Further distribution of this work must maintain attribution to the author(s) and the published article's title, journal citation, and DOI.

Supplement DOI: https://doi.org/10.6084/m9.figshare.16708729

Parent Article DOI: https://doi.org/10.1364/BOE.438400

Foveal-peripheral simultaneous aberrations with accommodation in myopic and emmetropic eyes: supplemental document

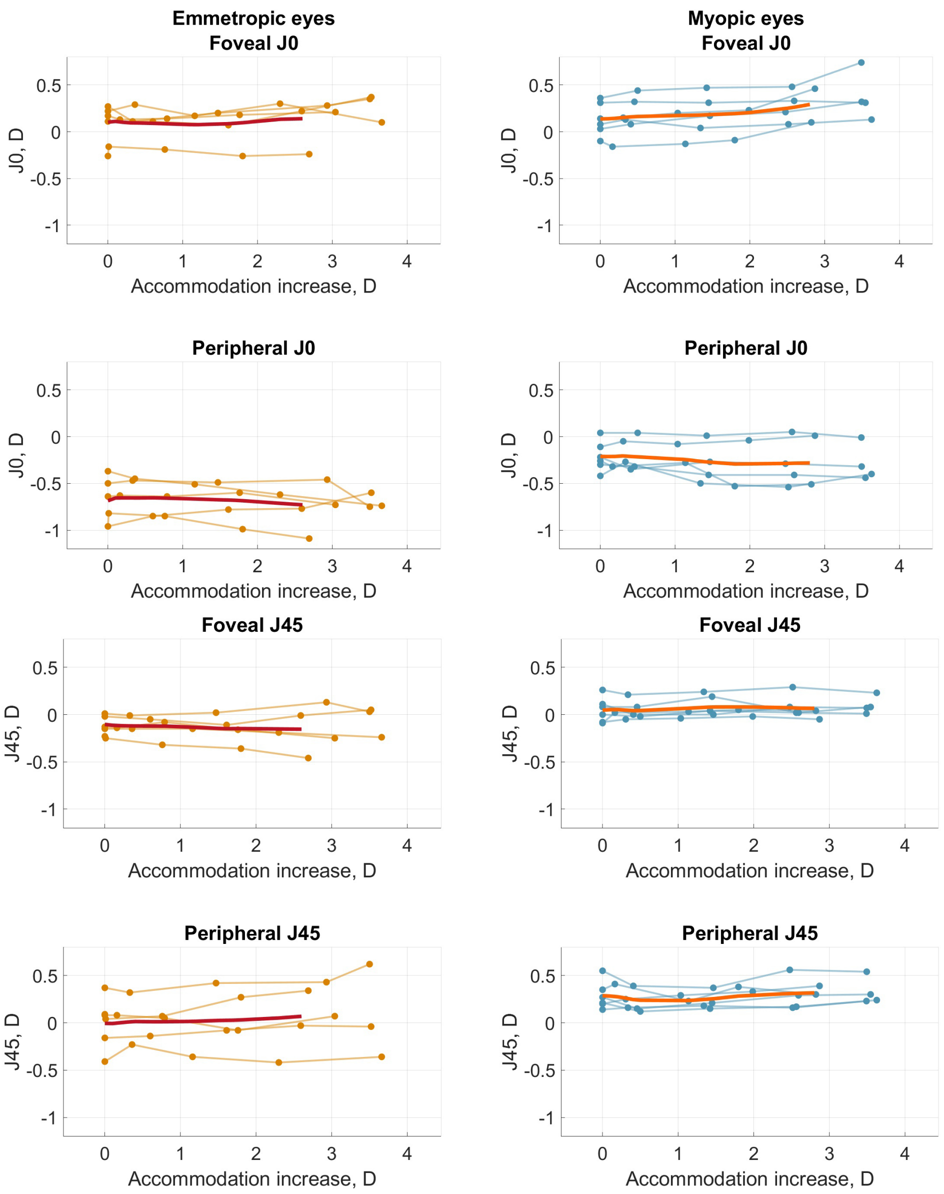

Fig. S1. Foveal and peripheral astigmatism as a function of accommodation increase for fovea and 20° nasal VF. Left column represents emmetropic group, and right column represents myopic group with spectacle correction. Thin lines correspond to individual eyes, dots show the measured values, and bold lines show average curves.

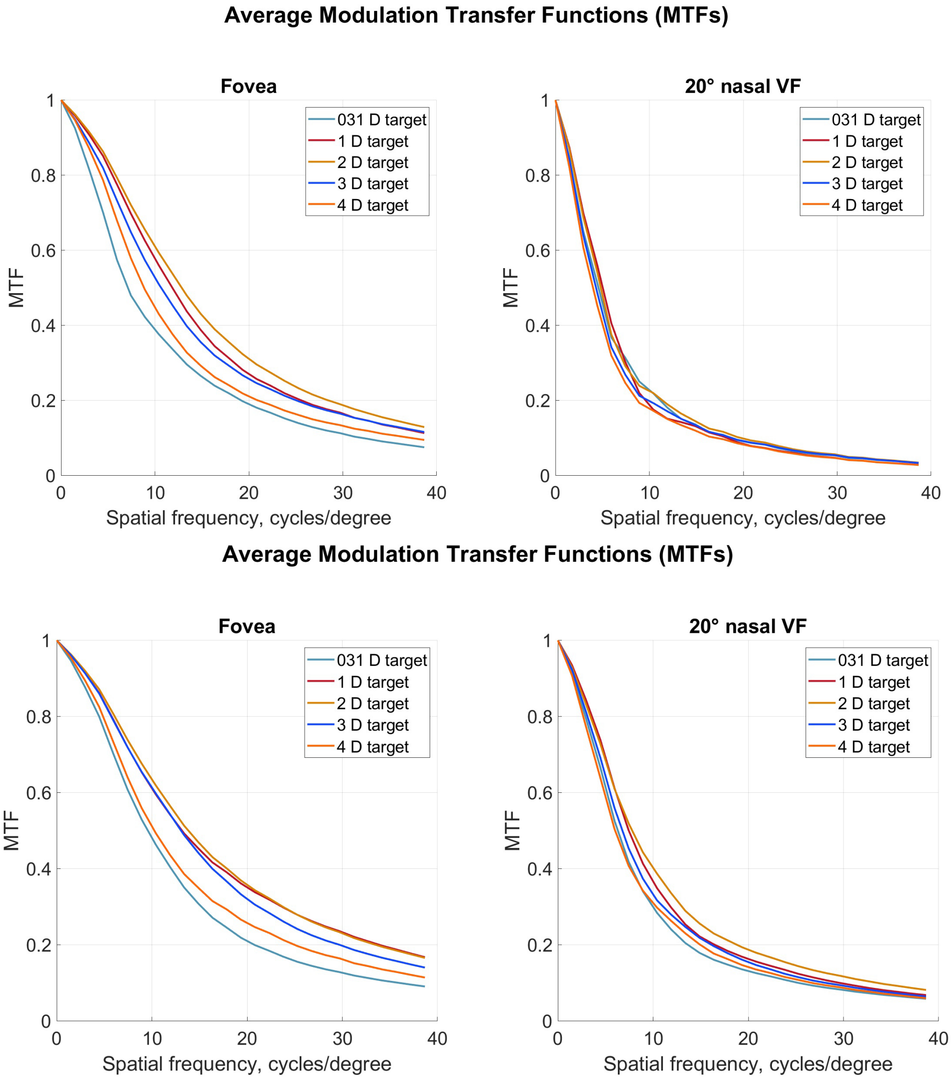

Fig. S2. AveragemModulation transfer functions for fovea and 20° nasal VF for emmetropic (**Top**, n = 5) and myopic (**Bottom**, n = 6) eyes with spectacle correction. The curves are calculated using Zernike coefficients up to the 6th order for wavelength 550 nm and 3.0 mm pupil diameter. Effect of the target vergence was compensated prior to the calculations.

Table S1. Average measured SE for myopic and emmetropic eyes for each target vergence. The table summarizes data depicted in Figure 1, left of the main manuscript. Note that the standard deviation here is between the participants (not related to AMFs).

**Target vergence, D**	-0.31	-1.0	-2.0	-3.0	-4.0
**Myopic eyes, foveal SE, D**	-0.50 ± 0.33	-0.88 ± 0.24	-1.91 ± 0.21	-3.04 ± 0.20	-4.16 ± 0.24
**Emmetropic eyes, foveal SE, D**	-0.76 ± 0.26	-1.05 ± 0.13	-1.92 ± 0.12	-3.04 ± 0.23	-4.04 ± 0.17

Table S2. Wilcoxon signed rank test results for the differences in Zernike coefficients between 0.31 D and 4.0 D accommodation targets in emmetropic eyes. Zernike coefficients are given in μm for 3.0 mm pupil diameter and λ = 550 nm. Note that for n = 5 the lowest achievable p-value is 0.06 (critical value of 0). Additionally, for each coefficient the average ± standard deviation (SD) for 0.31 D and 4.0 D targets are given. Note that the standard deviation here shows the variations of the average values and is not related to AMFs.

Zernike term	Emmetropic eyes, n = 5					
	Fovea			20° nasal VF		
	p-value	Average for 0.31 D	Average for 4.0 D	p-value	Average for 0.31 D	Average for 4.0 D
C_2^{-2}	0.44	+0.044 ± 0.030	+0.060 ± 0.060	0.63	+0.007 ± 0.100	−0.007 ± 0.122
C_2^{0}	0.06	+0.269 ± 0.114	+1.331 ± 0.019	0.06	+0.369 ± 0.114	+1.409 ± 0.155
C_2^{2}	0.63	−0.057 ± 0.076	−0.058 ± 0.071	0.19	+0.255 ± 0.077	+0.291 ± 0.047
C_3^{-3}	0.63	−0.025 ± 0.026	−0.033 ± 0.039	0.44	−0.003 ± 0.025	+0.008 ± 0.042
C_3^{-1}	0.44	+0.012 ± 0.015	+0.016 ± 0.024	0.63	+0.014 ± 0.016	+0.019 ± 0.035
C_3^{1}	0.81	+0.007 ± 0.011	+0.008 ± 0.013	0.63	−0.046 ± 0.036	−0.058 ± 0.057
C_3^{3}	0.13	+0.010 ± 0.017	+0.001 ± 0.022	0.13	−0.017 ± 0.035	−0.037 ± 0.03
C_4^{-4}	0.06	−0.002 ± 0.005	−0.012 ± 0.004	0.63	−0.001 ± 0.003	−0.003 ± 0.014
C_4^{-2}	0.31	−0.001 ± 0.005	−0.005 ± 0.012	0.06	+0.001 ± 0.009	+0.008 ± 0.014
C_4^{0}	1.00	+0.005 ± 0.005	+0.004 ± 0.011	1.00	+0.002 ± 0.01	+0.002 ± 0.018
C_4^{2}	0.12	−0.002 ± 0.005	+0.002 ± 0.008	0.81	−0.008 ± 0.01	−0.008 ± 0.017
C_4^{4}	0.06	+0.001 ± 0.006	+0.010 ± 0.011	0.63	+0.003 ± 0.01	+0.005 ± 0.005

Table S3. Wilcoxon signed rank test results for the differences in Zernike coefficients between 0.31 D and 4.0 D accommodation targets in myopic eyes with spectacle correction. Zernike coefficients are given in μm for 3.0 mm pupil diameter and λ = 550 nm. Statistically significant differences ($p \leq 0.05$) are marked with bold font. Additionally, for each coefficient the average ± standard deviation (SD) for 0.31 D and 4.0 D targets are given. Note that the standard deviation here shows the variations of the average values and is not related to AMFs.

Zernike term	Myopic eyes, spectacle correction, n = 6					
	Fovea			20° nasal VF		
	p-value	Average for 0.31 D	Average for 4.0 D	p-value	Average for 0.31 D	Average for 4.0 D
$C_2{-2}$	0.09	−0.010 ± 0.048	−0.024 ± 0.041	0.69	−0.112 ± 0.072	−0.107 ± 0.066
C_2^0	**0.03**	+0.191 ± 0.091	+1.348 ± 0.034	**0.03**	+0.169 ± 0.136	+1.388 ± 0.087
C_2^2	**0.03**	−0.057 ± 0.067	−0.106 ± 0.058	1.00	+0.090 ± 0.063	+0.093 ± 0.082
C_3^{-3}	0.06	−0.009 ± 0.019	−0.017 ± 0.025	0.16	−0.009 ± 0.022	+0.004 ± 0.029
C_3^{-1}	0.84	+0.007 ± 0.023	+0.007 ± 0.018	0.22	+0.007 ± 0.029	+0.023 ± 0.018
C_3^1	0.44	0.000 ± 0.022	+0.008 ± 0.014	0.09	−0.054 ± 0.032	−0.071 ± 0.026
C_3^3	0.44	−0.003 ± 0.015	0.000 ± 0.014	**0.03**	−0.018 ± 0.024	−0.047 ± 0.019
C_4^{-4}	0.44	+0.002 ± 0.007	−0.002 ± 0.007	0.84	−0.001 ± 0.012	−0.002 ± 0.003
C_4^{-2}	0.44	+0.003 ± 0.006	+0.001 ± 0.003	0.09	+0.005 ± 0.008	+0.008 ± 0.007
C_4^0	0.09	+0.008 ± 0.015	−0.004 ± 0.013	**0.03**	+0.004 ± 0.008	−0.017 ± 0.011
C_4^2	0.06	0.000 ± 0.004	+0.011 ± 0.012	0.16	0.000 ± 0.005	−0.006 ± 0.008
C_4^4	0.09	−0.008 ± 0.009	−0.001 ± 0.008	0.84	0.000 ± 0.007	+0.001 ± 0.015

Table S4. Wilcoxon signed rank test for the difference in Zernike coefficients between no correction and spectacle correction in myopic eyes when viewing the target at 0.25 m. Zernike coefficients are given in μm for 3.0 mm pupil diameter and λ = 550 nm. Other details are as for the Table S3.

Zernike term	Myopic eyes, n = 6					
	Fovea			20° nasal VF		
	p-value	Average for no correction	Average for spectacle correction	p-value	Average for no correction	Average for spectacle correction
$C_2 - 2$	0.69	-0.014 ± 0.100	-0.024 ± 0.040	0.56	-0.114 ± 0.132	-0.107 ± 0.066
C_2^0	0.44	$+1.382 \pm 0.210$	$+1.348 \pm 0.034$	0.44	$+1.436 \pm 0.307$	$+1.388 \pm 0.087$
C_2^2	0.22	-0.071 ± 0.074	-0.106 ± 0.058	**0.03**	$+0.170 \pm 0.082$	$+0.093 \pm 0.082$
C_3^{-3}	0.69	-0.016 ± 0.020	-0.017 ± 0.025	0.31	-0.004 ± 0.021	$+0.004 \pm 0.029$
C_3^{-1}	0.31	$+0.016 \pm 0.017$	$+0.007 \pm 0.018$	0.34	$+0.015 \pm 0.022$	$+0.023 \pm 0.018$
C_3^1	0.06	$+0.004 \pm 0.015$	$+0.009 \pm 0.014$	0.63	-0.077 ± 0.026	-0.071 ± 0.026
C_3^3	1.00	0.000 ± 0.017	$+0.001 \pm 0.014$	0.16	-0.038 ± 0.022	-0.047 ± 0.019
C_4^{-4}	1.00	-0.001 ± 0.005	-0.001 ± 0.008	0.09	$+0.004 \pm 0.005$	-0.002 ± 0.003
C_4^{-2}	0.50	0.000 ± 0.005	$+0.001 \pm 0.003$	0.78	$+0.008 \pm 0.008$	$+0.008 \pm 0.008$
C_4^0	**0.03**	$+0.002 \pm 0.013$	-0.003 ± 0.013	**0.03**	-0.005 ± 0.010	-0.017 ± 0.011
C_4^2	0.13	$+0.005 \pm 0.006$	$+0.011 \pm 0.012$	0.44	-0.003 ± 0.009	-0.006 ± 0.008
C_4^4	0.06	-0.005 ± 0.007	-0.001 ± 0.008	0.63	$+0.003 \pm 0.008$	$+0.001 \pm 0.015$
